# Ryanodine Receptor 2 Contributes to Impaired Protein Localization in Cyclic Nucleotide-Gated Channel Deficiency

**DOI:** 10.1523/ENEURO.0119-19.2019

**Published:** 2019-06-27

**Authors:** Hongwei Ma, Fan Yang, Michael R. Butler, Jacob Rapp, Yun-Zheng Le, Xi-Qin Ding

**Affiliations:** 1 Department of Cell Biology, University of Oklahoma Health Sciences Center (OUHSC), Oklahoma City, Oklahoma 73104; 2 Department of Medicine, University of Oklahoma Health Sciences Center (OUHSC), Oklahoma City, Oklahoma 73104; 3 Department of Ophthalmology, University of Oklahoma Health Sciences Center (OUHSC), Oklahoma City, Oklahoma 73104; 4 Harold Hamm Diabetes Center, University of Oklahoma Health Sciences Center (OUHSC), Oklahoma City, Oklahoma 73104

**Keywords:** calcium channel, cone, endoplasmic reticulum, photoreceptor, retinal degeneration, ryanodine receptor

## Abstract

The photoreceptor cyclic nucleotide-gated (CNG) channel plays a pivotal role in phototransduction and cellular calcium homeostasis. Mutations in the cone photoreceptor CNG channel subunits CNGA3 and CNGB3 are associated with achromatopsia and cone dystrophies. CNG channel deficiency leads to endoplasmic reticulum (ER) stress-associated cone apoptosis, protein mislocalization, and ER calcium dysregulation. This work investigated the potential mechanisms of protein mislocalization associated with ER calcium dysregulation using *Cnga3^-/-^* mice lacking ER Ca^2+^ channel ryanodine receptor 2 (RyR2) specifically in cones. Deletion of *Ryr2* improved outer segment (OS) localization of the cone proteins M-opsin, S-opsin, and cone phosphodiesterase subunit α′ (PDE6C) and decreased inner segment localization. One-month-old *Cnga3^-/-^* mice showed ∼30% of M-opsin, 55% of S-opsin, and 50% of PDE6C localized to the OS. *Cnga3^-/-^* mice with *Ryr2* deletion at the same age showed almost 60% of M-opsin, 70% of S-opsin, and 70% of PDE6C localized to the OS. Deletion of *Ryr2* nearly completely reversed elevations of the ER stress markers phospho-IRE1α and phospho-eIF2α and suppressed cone apoptosis. Consistent with the improved cone protein localization and reduced ER stress/cone apoptosis, cone survival was improved by deletion of *Ryr2*. The number of cones was increased by ∼28% in 2- to 4-month-old *Cnga3^-/-^* mice with *Ryr2* deletion compared with age-matched *Cnga3^-/-^* mice. This work demonstrates a role of RyR2/ER calcium dysregulation in protein mislocalization, ER stress, and cone death. The findings provide novel insights into the mechanisms of photoreceptor degeneration and support strategies targeting ER calcium regulation to manage retinal degeneration.

## Significance Statement

The cyclic nucleotide-gated (CNG) channel plays a pivotal role in phototransduction and photoreceptor calcium homeostasis. Mutations in the cone CNG channel subunits are associated with achromatopsia and cone dystrophies. CNG channel deficiency leads to endoplasmic reticulum (ER) stress-associated cone apoptosis, protein mislocalization, and ER calcium dysregulation. This work demonstrates a role of ER Ca^2+^ channel ryanodine receptor 2 and ER calcium dysregulation in protein mislocalization and subsequent ER stress and cone cell death. The findings provide novel insights into the mechanisms of photoreceptor degeneration. Altered calcium signaling and ER stress-associated cell death are common throughout retinal degenerative diseases. Thus, strategies targeting ER calcium regulation may help treat or slow photoreceptor degeneration.

## Introduction

The cone photoreceptor cyclic nucleotide-gated (CNG) channel plays a pivotal role in phototransduction. In darkness/dim light, the channel is kept open by the binding of cyclic guanosine monophosphate (cGMP), permitting the influx of Ca^2+^ and Na^+^ necessary to maintain the dark current. Light induces hydrolysis of cGMP by photoreceptor phosphodiesterase 6 (PDE6), resulting in closure of the channel, hyperpolarization of the cell, and phototransduction (Bonigk et al., 1993; Kaupp and Seifert, 2002). Mutations in genes encoding the channel subunits CNGA3 and CNGB3 account for ∼80% of all cases of achromatopsia and are associated with progressive cone dystrophies (Kohl et al., 1998; Nishiguchi et al., 2005). These diseases are characterized by severely impaired daylight vision, lack of color discrimination, photophobia, and slow progressing degeneration of cones.

Mice lacking functional CNG channels mimic phenotypes in human patients, displaying impaired cone function and progressive cone degeneration (Biel et al., 1999; Ding et al., 2009). Mechanistic studies show that cone death in CNG channel-deficient mice involves endoplasmic reticulum (ER) stress-associated apoptosis (Thapa et al., 2012; Ma et al., 2013) and impaired protein localization. These mice show reduced opsin localization to the cone outer segment (OS) and increased opsin localization to the inner segment (IS) and other photoreceptor regions (Michalakis et al., 2005; Xu et al., 2011). Gene supplementation of the channel subunits resumed cone opsin OS localization, light response, and photoreceptor survival (Michalakis et al., 2010; Carvalho et al., 2011). Phototransduction proteins are synthesized/processed in the IS where ER is located and translocated to the OS where they function. Aberrant protein trafficking and localization has been associated with photoreceptor degeneration (Bales and Gross, 2016; Agrawal et al., 2017).

The CNG channel is essential for cellular calcium homeostasis (Hackos and Korenbrot, 1999; Krizaj and Copenhagen, 2002). Measurements of intracellular Ca^2+^ levels showed reduced cytoplasmic Ca^2+^ in cones of CNG channel-deficient mice (Butler et al., 2017). The cellular calcium perturbation was also supported by the remarkable elevation of cellular cGMP levels in these mice (Xu et al., 2013), because cytosolic Ca^2+^ level negatively regulates biosynthesis of cGMP (Olshevskaya et al., 2002; Baehr and Palczewski, 2009). The Ca^2+^ measurements also showed altered cellular Ca^2+^ dynamics following ER store depletion (Butler et al., 2017). ER calcium homeostasis is primarily regulated by the two ER Ca^2+^ channels, the ryanodine receptor (RyR) and inositol-1,4,5-trisphosphate receptor (IP3R), for Ca^2+^ efflux out of the ER into the cytosol, and the sarco/endoplasmic reticulum Ca^2+^-ATPase, for Ca^2+^ influx into the ER. ER Ca^2+^ channels are highly regulated by cytosolic Ca^2+^ levels; their activity is increased when cytosolic Ca^2+^ level is low. In photoreceptors these channels are on the ER within the IS/perikaryon regions and regulate induction of phototransduction and tonic release of the photoreceptor neurotransmitter glutamate (Krizaj and Copenhagen, 2002; Krizaj, 2012). Photoreceptors express all three isoforms of RyR; RyR1, RyR2, and RyR3. RyR2 is the predominant form (Shoshan-Barmatz et al., 2007; Butler et al., 2017) and is distributed mainly in the IS of the photoreceptors and outer nuclear layer (Krizaj et al., 2003; Huang et al., 2011; Butler et al., 2017). Consistent with observed cytosolic calcium perturbation, CNG channel- deficient retinas show potential ER calcium dysregulation and impaired protein processing, manifested as elevated expression/activity of RyR and IP3R (Thapa et al., 2012; Butler et al., 2017) and elevated activity of ER stress/ER-associated protein degradation markers (Ma et al., 2015). Moreover, treatment with ER Ca^2+^ channel inhibitors reduced ER stress and cone death in these mice (Butler et al., 2017). This work investigated the effects of *Ryr2* deletion on cone protein localization and ER stress. The OS localization of cone opsins and PDE6C was significantly increased in *Cnga3^-/-^* mice lacking *Ryr2* and IS localization was significantly decreased, which were accompanied with reduced ER stress/cone death and improved cone survival. Our findings support the view that RyR2 function/ER calcium deficiency contributes to protein mislocalization and ER stress in CNG channel deficiency.

## Materials and Methods

### Mice, antibodies, and other reagents

The *Cnga3^-/-^* (Biel et al., 1999), *Hrgp-cre* [transgenic mice expressing Cre recombinase directed by the human red/green pigment (HRGP) gene promoter; Le et al., 2004], and *Nrl^-/-^* (Mears et al., 2001) mouse lines were generated as described previously. The *Ryr2^flox/flox^* (Bround et al., 2012) and wild-type (C57BL/6J) lines were obtained from The Jackson Laboratory (*Ryr2^flox/flox^*, JAX stock #026628; C57BL/6J, JAX stock #000664). The *Ryr2^flox/flox^/Hrgp-cre*, *Cnga3^-/-^/Ryr2^flox/flox^/Hrgp-cre*, *Cnga3^-/-^/Ryr2^flox/flox^/Hrgp-cre/Nrl^-/-^* (CR2N), and *Cnga3^-/-^/Nrl^-/-^* lines were generated by cross-breeding. All mice were housed under cyclic, 12 h light/dark conditions, with ∼7 foot candles of illumination during the light cycle. Animal maintenance and experiments were approved by the local Institutional Animal Care and Use Committee (OUHSC) and conformed to the guidelines on the care and use of animals accepted by the Society for Neuroscience and the Association for Research in Vision and Ophthalmology (Rockville, MD). Mice of either sex were used in the experiments.

Primary antibody information is listed in Table 1. Horseradish peroxidase-conjugated anti-rabbit secondary antibody was obtained from Kirkegaard & Perry Laboratories, fluorescent-conjugated goat anti-rabbit antibody was purchased from Invitrogen (A21428), Streptavidin-Cy3 was purchased from Sigma-Aldrich (S6402), and DAPI was purchased from Sigma-Aldrich (D9542). Other reagents were obtained from Sigma-Aldrich, Bio-Rad, Invitrogen, Abcam, and Tocris Biosciences.

**Table 1.** Primary antibody information

Antibody	Provider	Catalog no.	Dilutions used in immunoblotting (IB) or immunofluorescence labeling (IF)
M-opsin	MilliporeSigma	AB5405	1:200 (IF)
S-opsin	Dr. Muna Naash from the University of Houston		1:200 (IF)
PDE6C	Abgent	ap9728c	1:200 (IF)
Biotinylated PNA	Vector Laboratories	B-1075	1:200 (IF)
GFAP	DAKO	Z0334	1:500 (IF)
Phospho-eIF2α	Cell Signaling Technology, Beverly, MA	3398	1:500 (IB)
Phospho-IRE1a	Abcam, Inc., Cambridge, MA	ab-48187	1:500 (IB)
β-actin	Abcam, Inc., Cambridge, MA	ab-6276	1:2000 (IB)

### Eye preparation, immunofluorescence labeling, and confocal microscopy

Retinal whole mounts or cross sections were prepared for immunofluorescence labeling, as described previously (Ma et al., 2014). For retinal whole-mount preparations, eyes were enucleated, marked at the superior pole with a green dye, and fixed in 4% paraformaldehyde (Polysciences) for 30 min at room temperature, followed by removal of the cornea and lens. The eyes were then fixed in 4% paraformaldehyde in PBS for 4–6 h at room temperature, and retinas were isolated and the superior portion was marked for orientation with a small cut. For retinal cross-sections, mouse eyes were enucleated (the superior portion of the cornea was marked with green dye before enucleation) and fixed in Prefer (Anatech) for 25–30 min at room temperature. Paraffin sections (5 µm thickness) passing vertically through the retina (along the vertical meridian passing through the optic nerve head) were prepared using a Leica microtome (Leica Biosystems).

Immunofluorescence labeling was performed as described previously (Ma et al., 2014). Briefly, retinal whole mounts or sections were blocked with Hanks’ balanced salt solution containing 5% BSA and 0.5% Triton X-100 for 1 h at room temperature or overnight at 4°C. Prior to blocking, antigen retrieval was performed in 10 mM sodium citrate buffer, pH 6.0, in a 70°C water bath. Primary-antibody incubation was performed for 2 h at room temperature or overnight at 4°C (for antibody information, see Table 1). Slides were mounted and coverslipped after fluorescence-conjugated secondary-antibody incubation and wash steps. Immunofluorescence labeling was then imaged using an Olympus FV1000 confocal laser-scanning microscope and FluoView imaging software (Olympus). For evaluations of cone OS protein cellular localization, confocal images of 10 layers were stacked with the *Z*-stack function in the ImageJ software (https://imagej.nih.gov/ij/) to obtain a maximal immunofluorescence density. Fluorescence density levels of the immunolabeling in the OS, IS, outer nuclear layer (ONL), and outer plexiform layer (OPL) were measured, and the density levels at each region relative to the total fluorescence density were calculated and averaged from at least three sections per eye from at least four animals per condition.

### Scotopic and photopic electroretinography recordings

Full-field electroretinography (ERG) recordings were conducted as described previously (Xu et al., 2011). Briefly, after overnight dark adaptation, mice were anesthetized by intraperitoneal injection of 85 mg/kg ketamine and 14 mg/kg xylazine. ERGs were recorded using an Espion visual electrophysiology system with a Ganzfeld ColorDome system (Diagnosys). Potentials were recorded using a gold-wire electrode to contact the corneal surface through a layer of 2.5% hypromellose (Gonak, Akorn). For assessment of scotopic responses, a stimulus intensity of 2.20 log cd·s m^−2 ^was presented to dark-adapted dilated mouse eyes. To evaluate photopic responses, mice were adapted to a 1.48 log cd·s m^−2 ^light for 5 min, and then a light intensity of 1.89 log cd·s m^−2 ^was given. Responses were differentially amplified, averaged, and analyzed using Espion 100 software (Diagnosys).

### TUNEL assay

Terminal deoxynucleotidyl transferase dUTP nick-end labeling (TUNEL) was performed to analyze photoreceptor apoptotic death as described previously (Ma et al., 2015), using paraffin- embedded retinal sections and an *in situ* cell death fluorescein detection kit (Roche Applied Science, 11684795910). Immunofluorescence labeling was imaged using an Olympus FV1000 confocal laser-scanning microscope, and TUNEL-positive cells in the outer nuclear layer passing through the optic nerve were counted and averaged from at least three sections per eye from at least five animals per condition.

### Retinal protein preparation, SDS-PAGE, and Western blot analysis

Retinal protein preparation, SDS-PAGE, and Western blot analysis were performed as previously (Ma et al., 2015). Briefly, retinas were homogenized in homogenization buffer A (0.32 M sucrose, 20 mM HEPES, pH 7.4, and 3 mm EDTA containing protease and phosphatase inhibitors; Roche Applied Science, catalog #04693159001 and #04906837001, respectively), and homogenates were centrifuged at 3000 rpm for 10 min at 4°C. The resulting supernatant was then centrifuged at 13,000 rpm for 35 min at 4°C to separate cytosolic (supernatant) and membrane (pellet) fractions. The resulting membrane pellet was resuspended in homogenization buffer B (0.32 m sucrose, 20 mm HEPES, pH 7.4, 3 mm EDTA, and 0.1% Triton X-100 containing protease and phosphatase inhibitors, as previously described), sonicated twice for 15 s on ice at a medium speed using an XL2000 Ultrasonic Cell Disruptor (Misonix), with a 30 s recovery between disruptions, and incubated for 1 h at 4°C with gentle agitation. After incubation, the homogenate was centrifuged at 13,000 rpm for 35 min at 4°C, and the resulting supernatant was used as the membrane fraction. All protein concentrations were determined by a protein-assay kit from Bio-Rad Laboratories. Retinal protein samples (20 µg protein per sample) were then subjected to SDS-PAGE and transferred to PVDF membranes, which were subsequently blocked in 5% bovine serum albumin (BSA) for 1 h at room temperature. Immunoblots were incubated with primary antibody overnight at 4°C. After washing in Tris-buffered saline with 0.1% Tween 20, immunoblots were incubated with horseradish peroxidase-conjugated secondary antibody (1:20,000) for 1 h at room temperature. SuperSignal West Dura Extended Duration chemiluminescent substrate (ThermoFisher Scientific, catalog #34076) was used to detect binding of the primary antibodies to their cognate antigens. A Li-Cor Odyssey CLx Imager and Li-Cor software (Li-Cor Biosciences) were used for detection and densitometric analysis.

### Statistical analysis

One-way ANOVA and unpaired Student's *t* test were used to evaluate significant differences between multiple groups and two groups, respectively. Statistical analyses and graph generation were performed using GraphPad Prism software (GraphPad Software) for Windows.

## Results

### Deletion of *Ryr2* increased OS localization of cone opsins in Cnga3^-/-^ mice

**Figure 1.**
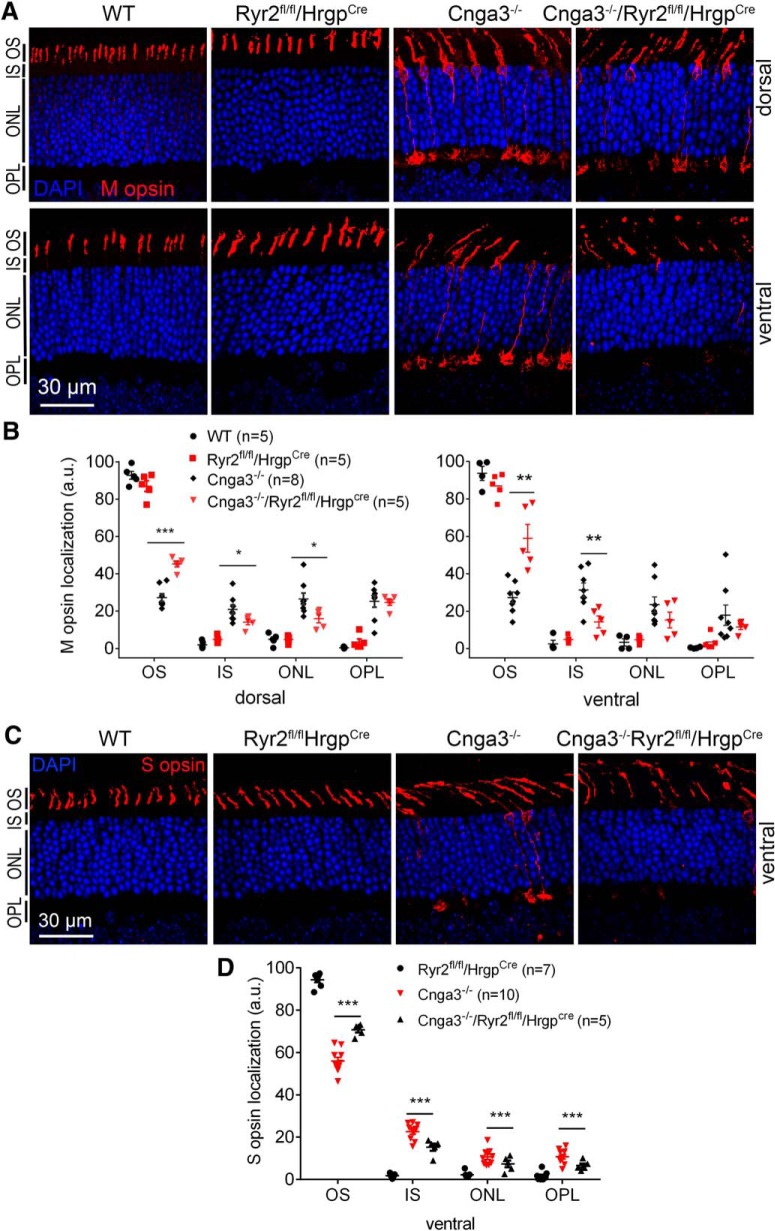
Deletion of *Ryr2* increased OS localization of cone opsin in *Cnga3^-/-^* mice. Cone opsin localization was evaluated by immunofluorescence labeling on retinal cross sections prepared from *Cnga3^-/-^/Ryr2^flox/flox^/Hrgp-cre*, *Cnga3^-/-^*, *Ryr2^flox/flox^/Hrgp-cre*, and wild-type (WT) mice at P30. ***A***, ***B***, Representative confocal images of immunofluorescence labeling of M-opsin (***A***), and corresponding quantitative analysis of immunofluorescence intensity level at different regions of the retinal cross sections (***B***). ***C***, ***D***, Representative confocal images of immunofluorescence labeling of S-opsin on ventral retina (***C***) and corresponding quantitative analysis of immunofluorescence intensity level at different regions of the retinal cross sections (***D***). Data are presented as mean ± SEM. *p < 0.05, **p < 0.01, ***p < 0.001.

Retinal cross-sections of *Cnga3^-/-^* mice show decreased levels of opsin localized to the cone OS and increased opsin levels in the cone IS, ONLs, and OPLs (Michalakis et al., 2005, 2010; Carvalho et al., 2011; Komáromy et al., 2011; Xu et al., 2011). In this study, we examined cone opsin localization in *Cnga3^-/-^* mice lacking *Ryr2* to determine the contribution of RyR2. *Cnga3^-/-^*, *Cnga3^-/-^/Ryr2^flox/flox^/Hrgp-cre*, *Ryr2^flox/flox^/Hrgp-cre*, and wild-type mice at postnatal day (P)30 were analyzed for M-opsin and S-opsin cellular localization by immunofluorescence labeling on retinal cross sections. The data from this study are presented in Figure 1. Deletion of *Ryr2* significantly increased M-opsin localization to the cone OS in *Cnga3^-/-^* mice and decreased its localization to the IS. The OS immunofluorescence density level of M-opsin labeling was ∼30% of the total immunofluorescence level in the ventral retina of *Cnga3^-/-^* mice, and it was ∼60% of the total level in *Cnga3^-/-^/Ryr2^flox/flox^/Hrgp-cre* mice (Fig. 1*A*,*B*). Similar improvement was observed in the dorsal region. The IS immunofluorescence density level was ∼30% of the total level in the ventral retina of *Cnga3^-/-^* mice, and it was ∼15% of the total level in *Cnga3^-/-^/Ryr2^flox/flox^/Hrgp-cre* mice (Fig. 1*A*,*B*). Similar observation was obtained in the dorsal region.

The significantly reduced immunofluorescence level of M-opsin labeling was also observed in the ONL and OPL regions. Similar to findings with M-opsin cellular localization, deletion of *Ryr2* significantly increased S-opsin localization to the OS in *Cnga3^-/-^* mice and decreased its localization to the IS. The OS immunofluorescence density level of S-opsin labeling was ∼55% of the total level in the ventral retina of *Cnga3^-/-^* mice, and it was ∼70% of the total level in *Cnga3^-/-^/Ryr2^flox/flox^/Hrgp-cre* mice (Fig. 1*C*,*D*). The IS immunofluorescence density level was ∼23% of the total level in the ventral retina of *Cnga3^-/-^* mice, and it was ∼15% of the total level in *Cnga3^-/-^/Ryr2^flox/flox^/Hrgp-cre* mice (Fig. 1*C*,*D*). The significantly reduced immunofluorescence level of S-opsin labeling was also observed in the ONL and OPL regions. In wild-type and *Ryr2^flox/flox^/Hrgp-cre* mice, M-opsin and S-opsin localized to the OS. There was no significant difference in opsin distribution between wild-type and *Ryr2^flox/flox^/Hrgp-cre* mice (Fig. 1).

### Deletion of *Ryr2* improved OS localization of PDE6C in Cnga3^-/-^ mice

**Figure 2.**
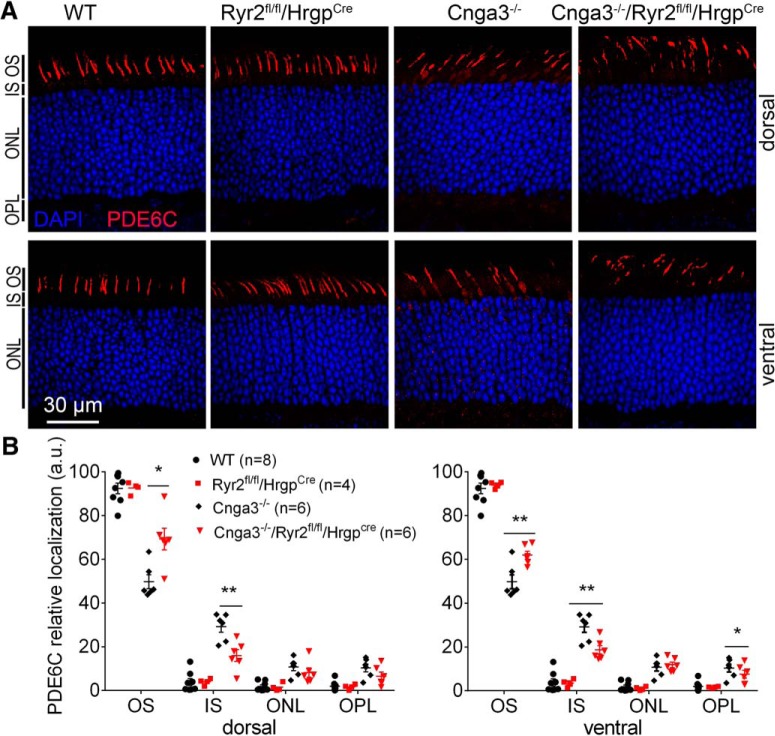
Deletion of *Ryr2* increased OS localization of PDE6C in *Cnga3^-/-^* mice. PDE6C localization was evaluated by immunofluorescence labeling on retinal cross sections prepared from *Cnga3^-/-^/Ryr2^flox/flox^/Hrgp-cre*, *Cnga3^-/-^*, *Ryr2^flox/flox^/Hrgp-cre*, and wild-type (WT) mice at P30. Shown are representative confocal images of immunofluorescence labeling of PDE6C (***A***) and corresponding quantitative analysis of immunofluorescence intensity level at different regions of the retinal cross sections (***B***). Data are presented as mean ± SEM. *p < 0.05, **p < 0.01.

We also examined cellular localization of the α′ subunit of PDE6 complex, PDE6C, in *Cnga3^-/-^* mice lacking *Ryr2*. *Cnga3^-/-^/Ryr2^flox/flox^/Hrgp-cre*, *Cnga3^-/-^*, *Ryr2^flox/flox^/Hrgp-cre*, and wild-type mice at P30 were analyzed for PDE6C cellular localization by immunofluorescence labeling on retinal cross sections. The data from this study are presented in Figure 2. In wild-type and *Ryr2^flox/flox^/Hrgp-cre* mice, PDE6C mainly localized to the OS. There was no detectable difference in PDE6C distribution between wild-type and *Ryr2^flox/flox^/Hrgp-cre* mice (Fig. 2). C*nga3^-/-^* mice showed apparent PDE6C mislocalization to the IS. Deletion of *Ryr2* significantly improved PDE6C localization to the OS and decreased its localization to the IS. The OS immunofluorescence density level of PDE6C labeling was ∼50% of the total immunofluorescence level in the ventral retina of *Cnga3^-/-^* mice, and it was ∼70% of the total level in *Cnga3^-/-^/Ryr2^flox/flox^/Hrgp-cre* mice. Similar improvement was observed in the dorsal region (Fig. 2). The IS immunofluorescence density level was ∼30% the total level in the ventral retina of *Cnga3^-/-^* mice, and it was ∼18% of the total level in *Cnga3^-/-^/Ryr2^flox/flox^/Hrgp-cre* mice. Similar observation was obtained in the dorsal region (Fig. 2).

### Deletion of *Ryr2* improved cone survival in Cnga3^-/-^ mice

**Figure 3.**
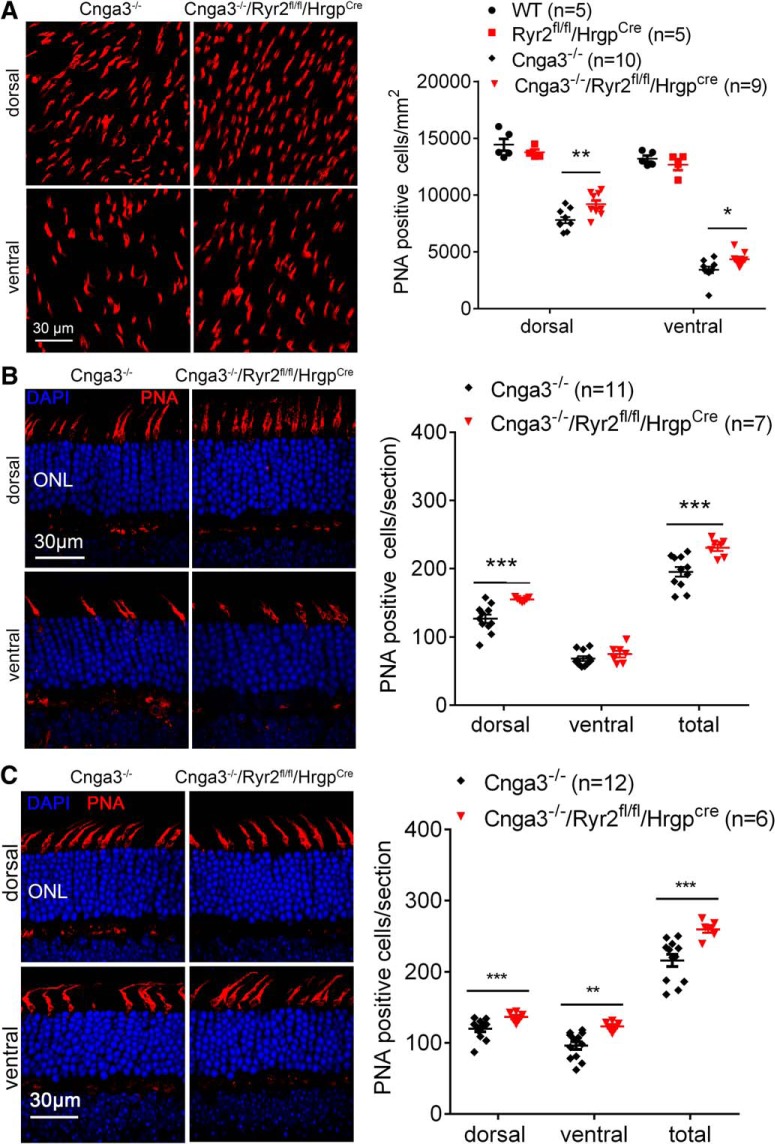
Deletion of *Ryr2* improved cone survival in *Cnga3^-/-^* mice. Cone density was evaluated by PNA immunofluorescence labeling on retinal whole mounts or retinal cross sections prepared from *Cnga3^-/-^/Ryr2^flox/flox^/Hrgp-cre*, *Cnga3^-/-^*, *Ryr2^flox/flox^/Hrgp-cre*, and wild-type (WT) mice. Shown are representative confocal images of PNA labeling on retinal whole mounts (***A***) and retinal cross sections (***B***) prepared from mice at 4 months, and PNA labeling on retinal cross sections prepared from mice at 2 months (***C***), and corresponding quantitative analysis. Data are presented as mean ± SEM. *p < 0.05, **p < 0.01, ***p < 0.001.

CNG channel-deficient mice display early onset, progressive cone degeneration with more severe degeneration in the ventral retina (Michalakis et al., 2005; Xu et al., 2011). We examined whether deletion of *Ryr2* improves cone survival. *Cnga3^-/-^/Ryr2^flox/flox^/Hrgp-cre*, *Cnga3^-/-^*, *Ryr2^flox/flox^/Hrgp-cre*, and wild-type mice at 4 months of age were examined for cone density by peanut agglutinin (PNA) labeling on retinal whole mounts and retinal cross sections. The data from this evaluation are presented in Figure 3. Deletion of *Ryr2* significantly increased cone density in *Cnga3^-/-^* mice. Evaluations on retinal whole mounts showed that the number of PNA-positive cells in the ventral and dorsal retina was increased by ∼28 and 17%, respectively, in *Cnga3^-/-^/Ryr2^flox/flox^/Hrgp-cre* mice compared with age-matched *Cnga3^-/-^* controls (Fig. 3*A*). There was no difference in the number of PNA-labeled cones between *Ryr2^flox/flox^/Hrgp-cre* and wild-type mice (Fig. 3*A*). Similar results were obtained from evaluations on retinal cross sections, showing cone density was increased by ∼26% in *Cnga3^-/-^/Ryr2^flox/flox^/Hrgp-cre* mice compared with *Cnga3^-/-^* controls (Fig 3*B*). In a separate experiment, we examined cone density in mice at 2 months and obtained similar findings. The number of PNA-positive cells in the ventral and dorsal retina was increased by ∼28 and 13%, respectively, in *Cnga3^-/-^ /Ryr2^flox/flox^/Hrgp-cre* mice compared with age-matched *Cnga3^-/-^* controls (Fig. 3*C*).

**Figure 4.**
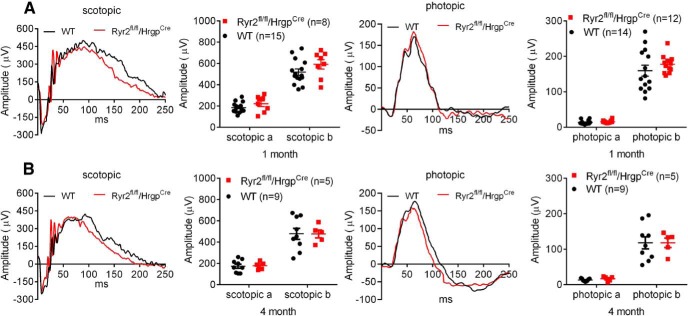
Deletion of *Ryr2* did not affect retinal light responses in wild-type mice. Retinal light responses in *Ryr2^flox/flox^/Hrgp-cre* and wild-type (WT) mice were evaluated by ERG analysis. Shown are representative scotopic and photopic ERG recording waves and quantification of the ERG recordings in *Ryr2^flox/flox^/Hrgp-cre* and WT mice at 1 (***A***) and 4 months (***B***). Data are presented as mean ± SEM.

To determine whether deletion of *Ryr2* in cones affects photoreceptor function, we performed ERG analysis in *Ryr2^flox/flox^/Hrgp-cre* and wild-type mice at 1 and 4 months. The evaluations showed that the a- and b-waves of the scotopic and photopic responses were not different between *Ryr2^flox/flox^/Hrgp-cre* and wild-type mice (Fig. 4), indicating that deletion of *Ryr2* in cones did not affect photoreceptor function. *Cnga3^-/-^* mice at 1 month displayed normal scotopic responses but no photopic responses (data not shown), as reported previously (Biel et al., 1999).

### Deletion of *Ryr2* reduced ER stress and cone apoptosis in CNG channel-deficient mice

**Figure 5.**
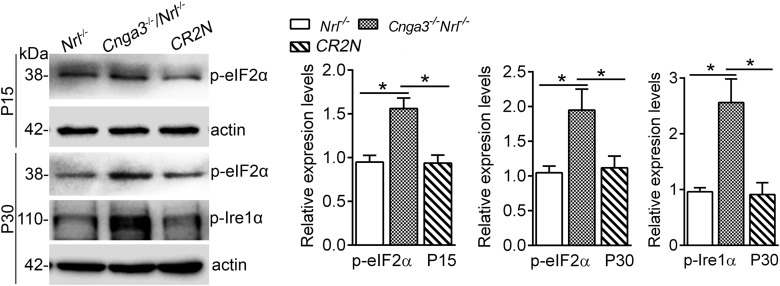
Deletion of *Ryr2* reduced ER stress in retinas of *Cnga3^-/-^*/*Nrl^-/-^* mice. Expression levels of ER stress marker proteins phospho-IRE1α and phospho-eIF2α in retinas of *Cnga3^-/-^/Ryr2^flox/flox^/Hrgp-cre/Nrl^-/-^* (CR2N), *Cnga3^-/-^/Nrl^-/-^*, and *Nrl^-/-^* mice at P15 and P30 were analyzed by Western blot analysis. Shown are representative Western blot images of these detections and corresponding quantitative analysis, following normalization to internal loading control β-actin. Data are presented as mean ± SEM of three independent assays using retinas from 6 to 8 mice. *p < 0.05.

Cone death in CNG channel-deficient mice involves ER stress-associated apoptosis (Thapa et al., 2012; Ma et al., 2013, 2015). Retinas of CNG channel-deficient mice display ER stress, characterized by the activation of the eukaryotic initiation factor 2α (eIF2α) and activation of the serine/threonine-protein kinase/endoribonuclease 1α (IRE1α; Thapa et al., 2012; Ma et al., 2015). This work examined effects of *Ryr2* deletion on the ER stress and cone apoptosis. *Cnga3^-/-^/Ryr2^flox/flox^/Hrgp-cre/Nrl^-/-^* (CR2N) line with deficiency of *Cnga3* and *Ryr2* on a cone (or cone-like)-dominant retina background (*Nrl^-/-^* background) was generated to evaluate ER stress in cones. Mice lacking NRL, a rod-specific neural retina leucine zipper transcription factor, confer a cone-dominant retina (Mears et al., 2001). We have previously shown that *Cnga3^-/-^/Nrl^-/-^* mice display loss of cone function and cone degeneration similar to *Cnga3^-/-^* mice (Thapa et al., 2012; Ma et al., 2013, 2015). Retinas of CR2N, *Cnga3^-/-^ /Nrl^-/-^,* and *Nrl^-/-^* mice at P15 and P30 were evaluated for levels of ER stress markers. We found that deletion of *Ryr2* significantly reduced ER stress. The elevated levels of phospho-IRE1α and phospho-eIF2α were nearly completely reversed in CR2N retinas compared with age-matched *Cnga3^-/-^/Nrl^-/-^* retinas (Fig. 5).

**Figure 6.**
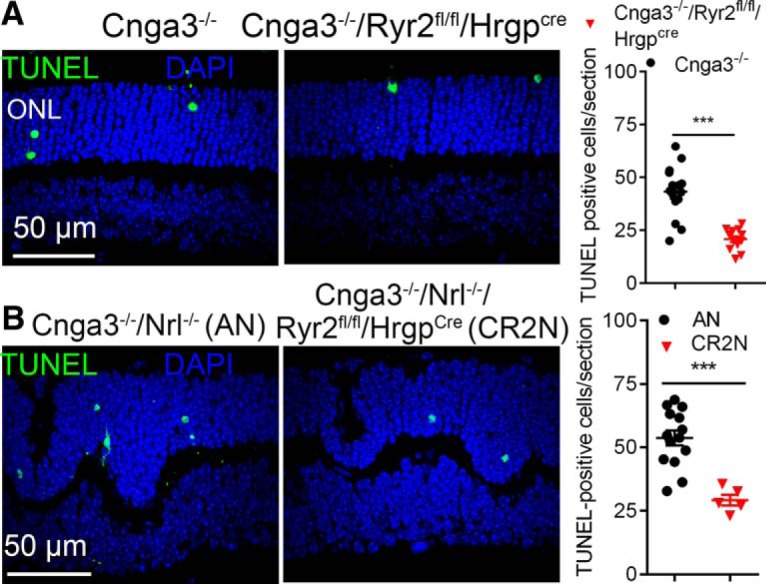
Deletion of *Ryr2* reduced cone apoptosis in *Cnga3^-/-^* and *Cnga3^-/-^*/*Nrl^-/-^* mice. Photoreceptor apoptosis was evaluated by TUNEL labeling on retinal cross sections of CNG channel-deficient mice at P15. Shown are representative confocal images of TUNEL labeling and correlating quantitative analysis in *Cnga3^-/-^/Ryr2^flox/flox^/Hrgp-cre* and *Cnga3^-/-^* mice, and corresponding quantitative analysis (***A***), and in *Cnga3^-/-^/Ryr2^flox/flox^/Hrgp-cre*/*Nrl^-/-^* (CR2N) and *Cnga3^-/-^/Nrl^-/-^* mice, and corresponding quantitative analysis (***B***). Data are represented as mean ± SEM. ***p < 0.001.

Effects of *Ryr2* deletion on cone apoptosis were evaluated by TUNEL. We found that *Ryr2* deletion significantly reduced cone death in *Cnga3^-/-^* and *Cnga3^-/-^/Nrl^-/-^* mice. The number of TUNEL-positive cells in *Cnga3^-/-^/Ryr2^flox/flox^/Hrgp-cre* mice at P15 was reduced by ∼46% compared with that in *Cnga3^-/-^* mice (Fig. 6*A*). Similar results were obtained in CR2N mice. The number of TUNEL-positive cells in CR2N mice was reduced by ∼52% compared with that in *Cnga3^-/-^/Nrl^-/-^* mice (Fig. 6*B*).

### Deletion of *Ryr2* in cones reduced activation of Müller cells in Cnga3^-/-^ mice

**Figure 7.**
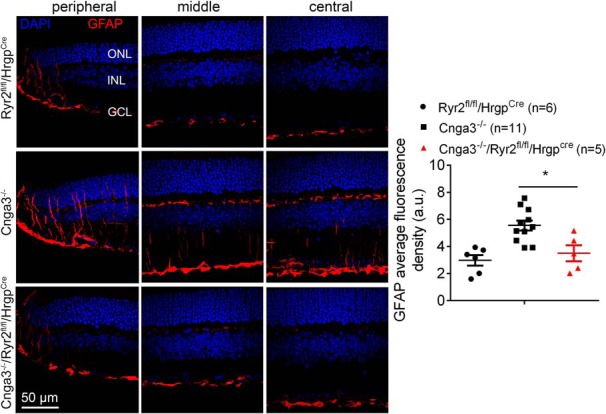
Deletion of *Ryr2* reduced activation of Müller glial cells in *Cnga3^-/-^* mice. GFAP immunofluorescence labeling was performed on the retinal cross sections prepared from *Cnga3^-/-^/Ryr2^flox/flox^/Hrgp-cre*, *Cnga3^-/-^*, and *Ryr2^flox/flox^/Hrgp-cre* mice at P30. Shown are representative confocal images of immunofluorescence labeling of GFAP on the peripheral, middle, and central regions of the retinal sections and corresponding quantification of immunofluorescence intensity. RGC, Retinal ganglion cell. Data are presented as mean ± SEM. *p < 0.05.

Müller glia are known to activate in response to retinal stress, including photoreceptor degeneration, by profound upregulation of glial fibrillary acidic protein (GFAP) in intermediate filaments. GFAP expression was previously shown to be increased in CNG channel-deficient retinas (Michalakis et al., 2005; Ma et al., 2015). In this study, we examined GFAP expression in *Cnga3^-/-^* mice with *Ryr2* deletion. *Cnga3^-/-^/Ryr2^flox/flox^/Hrgp-cre*, *Cnga3^-/-^*, *Ryr2^flox/flox^/Hrgp-cre*, and wild-type mice at P30 were evaluated for expression of GFAP by immunolabeling on retinal sections. We found that deletion of *Ryr2* significantly reduced expression of GFAP in *Cnga3^-/-^* mice. The retinal sections from *Cnga3^-/-^* mice showed significant upregulation of GFAP, with immunostaining mainly detected in the inner plexiform layers and the ganglion cell layer (Fig. 7), and this elevation was completely reversed by deletion of *Ryr2* with expression levels of GFAP comparable to those in *Ryr2^flox/flox^/Hrgp-cre* mice (Fig. 7). The GFAP labeling in *Ryr2^flox/flox^/Hrgp-cre* mice was not different from that in wild-type mice (data not shown).

## Discussion

Cones in *Cnga3*^-/-^ mice display characteristics of protein mislocalization, ER stress-associated apoptosis, and cellular/ER calcium dysregulation/deficiency. As a Ca^2+^ store, the ER is the site of protein folding/processing, and ER calcium homeostasis is crucial for protein folding and subsequent trafficking (Duncan et al., 2010; Mekahli et al., 2011). We hypothesized a role of ER Ca^2+^ deficiency in protein mislocalization in photoreceptor degeneration and investigated the effects of *Ryr2* deletion. We show that OS localization of cone opsins and PDE6C was significantly increased in *Cnga3^-/-^/Ryr2^flox/flox^/Hrgp-cre* mice compared with *Cnga3^-/-^* mice, IS localization of these proteins were significantly decreased, and the localization improvement was accompanied with reduced ER stress, improved cone survival, and reduced retinal stress/remodeling. Our findings demonstrate a role of RyR2/ER calcium dysregulation in protein mislocalization, ER stress and cone death in CNG channel deficiency. Deletion of *Ryr2* did not affect cone function, survival, and protein localization in wild-type mice, suggesting that healthy cones are not sensitive to lack of RyR2 (at least at the ages examined). It should be mentioning that deletion of *Ryr2* did not cause compensatory elevation of expression of IP3R or other isoforms of RyR (Bround et al., 2012).

### Deletion of *Ryr2* is sufficient to improve OS localization of cone proteins

Although all three isoforms of RyR are expressed in mouse retinas RyR2 is the predominant form expressed (Shoshan-Barmatz et al., 2007; Butler et al., 2017). Deletion of *Ryr2* improved protein OS localization, suggesting that targeting RyR2 is sufficient to improve ER calcium homeostasis and protein processing. It should be pointed out that deletion of *Ryr2* leads to only partial rescue of protein OS localization, suggesting an incomplete correction of ER Ca^2+^ deficiency. There are several possible explanations. First, as shown previously, ER calcium dysregulation in *Cnga3^-/-^* mice is also contributed by IP3R activity (Butler et al., 2017). The elevated IP3R function (in response to reduced cytosolic Ca^2+^ and elevated cGMP/PKG signaling) continues dysregulating ER calcium homeostasis in *Cnga3^-/-^* mice with *Ryr2* deletion. Second, there are other ER calcium/protein folding regulators, including ER shaping proteins (Zhang and Hu, 2016; Agrawal et al., 2017) and chaperones (Hartl et al., 2011; Saibil, 2013). These factors might have been involved in the impaired protein processing in CNG channel deficiency. Third, there are other Ca^2+^ channels in the IS membranes, including stromal interaction molecule proteins and transient receptor potential channels (Baldridge et al., 1998; Szikra et al., 2008). These channels might have been involved in the cytosolic/ER calcium dysregulation in CNG channel deficiency. Moreover, although *Cnga3^-/-^* cones experience ER calcium dysregulation, this may not be the only problem that these cells experience. For instance, CNG channel deficiency results in malformation of cone OS (Biel et al., 1999; Ding et al., 2009). This could also be a possible reason for incomplete rescue in protein localization.

### RyR2 function/ER calcium dysregulation represents a potential mechanism of protein mislocalization in photoreceptors

Photoreceptors are highly polarized neurons with cilia structure. Proper trafficking and location to the OS of the signaling and structural proteins is essential for photoreceptor functioning. Protein mislocalization is commonly observed in animal models of photoreceptor degenerative diseases. There are two well known mechanisms. One is because of the defects of the trafficking machinery, such as the ciliary protein centrosomal protein of 290 kDa (CEP290), retinitis pigmentosa GTPase regulator (Rachel et al., 2012a,b), and Arf-like protein 3 (Hanke-Gogokhia et al., 2016). The other is related to the mutations of photoreceptor proteins that interfere with folding of the proteins, such as S334ter and P23H rhodopsin mutants (Concepcion and Chen, 2010; Gorbatyuk et al., 2010; Griciuc et al., 2010). This work showing improved protein OS localization after deletion of *Ryr2* demonstrates the contribution of RyR2 function/ER calcium dysregulation to protein mislocalization. The precise mechanism underlying how deletion of RyR2/inhibition of ER Ca^2+^ release improves protein localization remains to be elucidated. It is our general assumption/hypothesis that deletion of RyR2/inhibition of ER Ca^2+^ release may not affect protein trafficking directly. Instead, it likely improves ER protein processing/folding function, which is highly regulated by ER Ca^2+^ level and might be impaired in CNG channel-deficient mice; the improved protein processing in turn facilitates protein localization. The potentially impaired protein processing/ER protein homeostasis in CNG channel-deficient cones is supported by the following observations. (1) Treatment with the chemical chaperone tauroursodeoxycholic acid (Ma et al., 2013; Butler et al., 2017) and molecular chaperone 11-*cis*-retinal (our unpublished observations) reduced ER stress and cone apoptosis, and (2) CNG channel-deficient cones showed increased cleavage of the activating transcription factor 6 and increased phosphorylation of IRE1α (Ma et al., 2015; Butler et al., 2017), which reflect unfolded protein response (UPR) and increased ER-associated protein degradation (Hetz, 2012; Wang and Kaufman, 2012). Nevertheless, the effects of CNG channel deficiency on ER Ca^2+^ level and ER protein processing/folding function, and the consequences after deletion of ER Ca^2+^ channels merit further investigation.

### Deletion of *Ryr2* is sufficient to reduce ER stress/cone apoptosis and improve cone survival

Though protein localization was partially improved in *Cnga3^-/-^/Ryr2^flox/flox^/Hrgp-cre* mice, ER stress responses were nearly completely corrected in these mice and cone apoptosis was greatly reduced. These data suggest that targeting RyR2 is sufficient to correct ER stress and reduce cone apoptosis. ER calcium dysregulation and its contribution to ER stress/cone death in CNG channel-deficient mice has been supported by several findings. These mice show the elevated expression/activity of RyR2 and treatment with chemical inhibitors of RyR reduces ER stress and cone apoptosis (Butler et al., 2017). These mice also show elevated expression/activity of IP3R, and treatment with chemical inhibitors of IP3R reduces ER stress/cone apoptosis and increases cone opsin OS localization (Butler et al., 2017). Moreover, the ER stress-associated cone apoptotic death has been well documented in CNG channel-deficient mice. Retinas of these mice show elevation of all three arms of the ER stress pathways, i.e., elevated levels of phospho-eIF2α and phospho-IRE1α and increased cleavage of ATF6 (Thapa et al., 2012; Ma et al., 2015; Butler et al., 2017), as well as increased nuclear localization of CCAAT/-enhancer-binding protein homologous protein (Thapa et al., 2012; Ma et al., 2015). These mice also show up-regulation of the cysteine protease calpains and cleavage of caspase-12 and caspase-7 in the retina (Thapa et al., 2012).

### Potential mechanisms of protein mislocalization, ER stress, and cone death in CNG channel deficiency: involvement of ER calcium dysregulation (Fig. 8)

As nonselective cation channels, CNG channels are the main source of the Ca^2+^ inward currents in the OS of photoreceptors and play a pivotal role in the light response/adaptation and cellular calcium homeostasis. Cone photoreceptors lacking functional CNG channels experience cellular calcium perturbation/cytosolic Ca^2+^ reduction (Butler et al., 2017) and subsequently elevated cellular cGMP/PKG signaling (Xu et al., 2013). The reduced cytosolic Ca^2+^ level and elevated cGMP/PKG signaling leads to excessive ER Ca^2+^ release via activation of ER Ca^2+^ channels (Ma et al., 2015; Butler et al., 2017), which consequently leads to ER calcium dysregulation and interferes with protein processing/folding, resulting protein localization impairment and UPR/ER stress. Chemical or genetic targeting ER Ca^2+^ channels may represent a novel strategy to improve ER protein processing and relieve ER stress.

**Figure 8.**
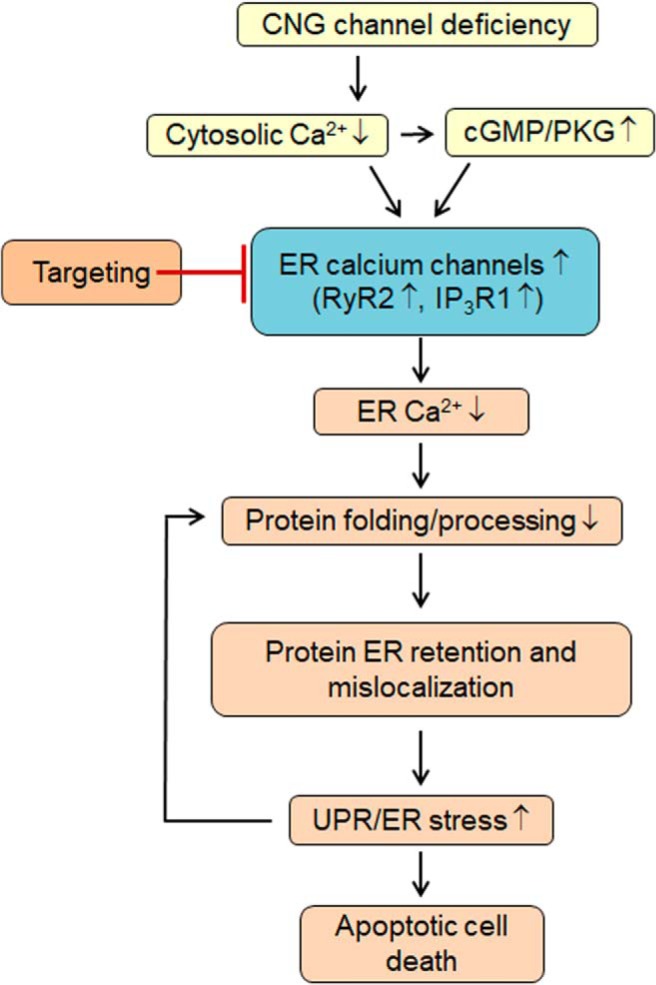
Potential mechanisms of protein mislocalization, ER stress, and cone death in CNG channel deficiency: involvement of ER calcium dysregulation. In *Cnga3^-/-^* cones, cytosolic Ca^2+^ is reduced, which increases cellular cGMP level and PKG signaling. Decreased cytosolic Ca^2+^ and increased cGMP/PKG signaling activates ER Ca^2+^ channels to remedy the cytosolic Ca^2+^ perturbation. As a consequence, the ER experiences calcium dysregulation/deficiency, which interferes with protein processing/folding and leads to protein ER retention and impaired localization. Furthermore, UPR/ER stress likely in turn further interferes with ER protein processing. Suppression of ER Ca^2+^ release by targeting ER Ca^2+^ channels retains Ca^2+^ in the ER, maintains the ER calcium homeostasis and protein processing/folding function, improves protein localization, and reduces ER stress/cone death.

In summary, the present study shows that deletion of *Ryr2* improved protein OS localization and suppressed ER stress/cone apoptosis. Moreover, deletion of *Ryr2* improved cone survival and reduced retinal stress/remodeling. The findings demonstrate a role of RyR2 function/ER calcium dysregulation in protein mislocalization and cellular stress/death in CNG channel deficiency, and provide novel insights into the mechanisms of photoreceptor degeneration. Because altered calcium signaling, protein processing impairment, and ER stress-associated cell death are common throughout retinal degenerative diseases, insights into these dynamic signaling pathways would help development of strategies targeting ER calcium regulation for photoreceptor protection.

## Synthesis

Reviewing Editor: Leonard Maler, University of Ottawa Decisions are customarily a result of the Reviewing Editor and the peer reviewers coming together and discussing their recommendations until a consensus is reached. When revisions are invited, a fact-based synthesis statement explaining their decision and outlining what is needed to prepare a revision will be listed below. The following reviewer(s) agreed to reveal their identity: Jonathan Lin. Note: If this manuscript was transferred from JNeurosci and a decision was made to accept the manuscript without peer review, a brief statement to this effect will instead be what is listed below.

Dear authors

both reviewers now agree that this latest version of your Ms has satisfied almost all of their points and is now potentially suitable for publication in eNeuro.

One reviewer did require some changes but these should be simple to implement.

I present the point below.

Please carefully make the corrections exactly as specified and include both a letter noting your edits and a redlined version. This will only require an editorial check but I will want to see this major point addressed,

R1: “The authors successfully addressed most of my critical comments, except for one. Despite clearly understanding the difference, they continue to confuse protein trafficking with protein localization. Trafficking is a well-defined process consisting of protein delivery from the biosynthetic membranes to their final location in the cell. Protein trafficking was not even studied in this investigation, only protein localization was. As the authors correctly point out in Discussion, all defects of protein localization they documented could be explained by these proteins accumulating in the ER/Golgi without any impairment of the actual trafficking mechanisms. So, unless the authors are willing to perform additional experiments addressing whether mislocalized proteins are actually delivered to a distinct cellular location other than the outer segment (e.g. the plasma membrane of the inner segment) and are not just stuck in the ER/Golgi (and perhaps in lysosomes as well), they should not describe this phenotype as a trafficking defect anywhere in the manuscript, including the title and the abstract. This is not a minor point as the current text is essentially misleading.”
